# The Effect of Micron-Sized TiB_2_ Particles on the Properties of Al6061 Strengthened with 4% TiB_2_ Nano-TiB_2_

**DOI:** 10.3390/ma17010182

**Published:** 2023-12-28

**Authors:** Xinbing Zheng, Wei Long, Changshun Zhu, Longbin Zhao, Xinbin Hu, Sheng Liu, Wenming Jiang, Yaxiong Peng

**Affiliations:** 1 School of Materials and Chemical Engineering, Hubei University of Technology, Wuhan 430068, China; 102110502@hbut.edu.cn (X.Z.); 102100466@hbut.edu.cn (C.Z.); zhaolongbin1997@163.com (L.Z.); huxbshu@163.com (X.H.); liusheng@hbut.edu.cn (S.L.); 17371991150@163.com (Y.P.); 2 Key Laboratory of Green Materials for Light Industry of Hubei Provincial, Wuhan 430068, China; 3 Hubei Engineering Laboratory of Automotive Lightweight Materials and Processing, Wuhan 430068, China; 4 State Key Laboratory of Material Processing and Die & Mould Technology, Huazhong University of Science and Technology, Wuhan 430074, China; wmjiang@hust.edu.cn

**Keywords:** powder metallurgy, high-energy ball milling, double-scale, TiB_2_, particle-reinforced

## Abstract

Dual-scale (nano and micron) particle-reinforced TiB_2_/6061Al matrix composites with different contents of TiB_2_ were prepared using powder metallurgy, and then analyzed via microstructure observation and tests of microhardness, tensile properties, and friction and wear properties. The 6061Al powders’ particles changed from spherical to flaky after two rounds of high-energy ball milling, and the TiB_2_ enhancer was embedded in or wrapped by the matrix particles after high-energy ball milling. Metallurgical bonding between TiB_2_ particles and the matrix was achieved, and Al_3_Ti was synthesized in situ during sintering. The hot-pressing process eliminated the internal defects of the composites, and the TiB_2_ particles were diffusely distributed in the matrix. The best comprehensive mechanical properties (hardness and tensile strength) were achieved when the mass fraction of TiB_2_ was 5% (1% micron + 4% nano); the hardness and tensile strength of the composites reached 131 HV and 221 MPa—79.5% and 93.9% higher than those of the pure matrix, respectively. The composites’ average coefficient of friction and volumetric wear rate were reduced. Composites with a TiB_2_ mass fraction of 7% (3% micron + 4% nano) had the highest average coefficients of friction and the lowest volumetric wear rate of 0.402 and 0.216 mm^3^∙N^−1^∙m^−1^, respectively. It was observed that adhesion influences the friction mechanism, which transitions from adhesive wear with slight oxidative wear to abrasive wear.

## 1. Introduction

Particle-reinforced aluminum matrix composite is a material with excellent comprehensive mechanical properties; it is created by adding particles to the aluminum alloy matrix externally, according to a specific process. With its light weight, high strength, high hardness, and wear resistance, it sees widespread use in automobile manufacturing, aerospace, electronic power, and other fields. It is the most mature variety of metal matrix composite materials [1,2,3,4]. The preparation of this material generally includes stir casting, in situ synthesis, extrusion casting, powder metallurgy, etc. [5,6,7,8,9]. Of these, powder metallurgy is an excellent composite preparation technology, offering flexible selection of reinforcement phase, precise and controllable content, strong designability, and reduced brittleness in the particle/matrix. The commonly used particle reinforcement phase sizes include micron, sub-micron, and nanometer [10]. The addition of a single micron particle reinforcement phase, achieved by adjusting the spatial distribution between the two phases, can improve the strength, hardness, and wear resistance of aluminum matrix composite, but greatly reduces its plastic toughness [11,12]. In addition, numerous studies have demonstrated that nano-scale reinforcement materials exhibit a better reinforcement effect than micron particles in aluminum matrix composites. However, nanoparticles have a large amount of surface energy and are easy to agglomerate, which has a negative impact on their reinforcement effect [13,14,15,16]. In recent years, due to the increasing demand for aluminum matrix composites with better comprehensive properties, researchers are no longer limited to single-scale particle reinforcement. Studies [16,17,18] have shown that incorporating micron-sized particles can efficiently prevent the agglomeration of nanoparticles, allowing for the optimal utilization of synergistic enhancement effects between micro- and nano-reinforced aluminum matrix composites. This ultimately enhances the properties of the aluminum matrix composites. Zhang et al. [19] prepared the (micron + nano) bimodal-sized SiCp/Al2014 composites using semi-solid stirring. These composites had yield strengths and failure strains of 358 MPa and 9.9%, respectively, which were superior to those of 2014Al composites reinforced by a single nano- or micrometer particle. An et al. [20] prepared Micro-B_4_C/nano-Ti hybrid particulate-reinforced copper matrix composites using high-energy ball milling (HEBM) and discharge plasma sintering (SPS) processes. The results revealed uniformly distributed (B_4_C + Ti) particles in the Cu matrix and a good interfacial bond between the reinforcement and the Cu matrix. The interface bonding mechanism comprised both metallurgical bonding and mechanical bonding. The mechanical properties (microhardness, tensile yield strength, ultimate tensile strength, and elongation to fracture) significantly improved compared to those of the pure copper.

Aluminum alloy, which contains trace elements, often performs better than pure aluminum, so it is often used in preference to pure aluminum as a matrix material. The six-series aluminum alloys are weaker than the two- and seven-series aluminum alloys, but their plasticity and toughness show significant improvement [21,22,23]. 6061Al, an excellent material, not only has the advantages of high strength, good corrosion resistance, and low cost, but also has excellent formability, weldability, and machinability [24,25]. Common additions to particle-reinforced aluminum matrix composites include TiC, SiC, B_4_C, Al_2_O_3_, TiO_2_, SiO_2_, ZrB_2_, TiB_2_, etc. TiB_2_ particles have a high melting point, high hardness, high elastic modulus, and good thermal stability. Additionally, the surface wettability of TiB_2_ by aluminum matrix is good and does not react with aluminum. This can prevent the generation of interfacial products, making it an ideal reinforcing phase [26]. Khoshy et al. [27] used stir casting to incorporate titanium diboride (TiB_2_) particles of different weight percentages (1, 3, and 5%) into Al6061. The results show that the hardness, strength, and wear resistance of Al6061-TiB_2_ composites increase as the weight percentage of TiB_2_ is increased, with the ultimate tensile strength of the composites increasing from 126.26 MPa to 290 Mpa. Suresh et al. [28] used high-energy stir casting for Al6061 aluminum alloy with different percentages of TiB_2_ (0, 2, 4, 6, 8, and 10%), and the mechanical properties, such as tensile strength, wear resistance, and hardness, increased with the percentage of TiB_2_ present in the samples when compared with the base aluminum alloy. Zhuang et al. [29] prepared TiB_2_/6061 in situ composites using high-energy ball milling and stir casting. The results showed that tensile strength, yield strength, Young’s modulus, and abrasion resistance all greatly improved, and the TiB_2_ particles had an average size of 1 μm and a polygonal shape. The average grain size of the composites was refined significantly as the TiB_2_ particle mass content increased from 1 to 3%; however, grain coarsening occurred in the 5 wt.% TiB_2_/6061 composites. The wear test results indicated that the average friction coefficient and wear rate of the TiB_2_/6061 composites initially increased and then decreased with the increase in the TiB_2_ content. Therefore, 6061Al was selected as the base material and TiB_2_ as the reinforcement phase.

In this study, dual-scale (micron, nano) TiB_2_ particle-reinforced aluminum matrix composites were prepared using powder metallurgy. The reinforcement was introduced via a direct addition method using two high-energy ball mills to change the powder configuration to flakes. The addition of Ti improved the wettability between the matrix and the reinforcement. In situ synthesis of Al_3_Ti occurred through the reaction of Ti with Al after sintering, further enhancing the strength and thermal stability of the composites [30,31,32,33]. Hot-pressing was then carried out to further improve the properties of the composites. Then, hot-pressing was performed to further improve the composite properties. The microstructure and morphology of the composite were analyzed, and the hardness, tensile strength, and friction and wear tests were conducted.

## 2. Methods and Materials

### 2.1. Materials for Double-Scale TiB_2_/(6061Al + Ti) Composites

The raw materials used in this experiment are as follows: base material 6061Al powder (purity > 99%, particle size < 500 mesh, Sinopsin Group Chemical Reagents Co., Ltd., Nanjing, China), alloy composition shown in Table 1; micron-grade TiB_2_ (<500 mesh) and nano-grade TiB_2_ (<50 nm) (purity > 99%, Beijing Dekedao Gold Technology Co., Ltd., Beijing, China), alloy composition shown in Table 2; Ti powder (purity > 99%, particle size < 300 mesh, MACKLIN, Shanghai, China).

**Figure 1:**
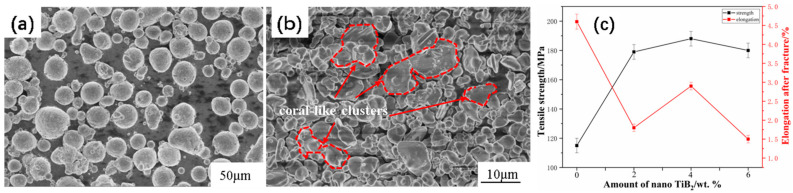
SEM images of experimental materials. (**a**) 6061Al powder; (**b**) TiB_2_ powder. (**c**) Tensile strength and elongation at break of nano TiB_2_/6061Al composites.

Figure 1 contains SEM images of the original powder. Figure 1a shows that the matrix material 6061Al powders are composed of spherical particles, while Figure 1b shows that TiB_2_ enhancer powders are composed of irregular particles partially agglomerated from coral-like clusters. In this study, the optimum amount of nanoparticles to be added was determined upfront, and tensile tests revealed 4% nano TiB_2_ as the optimal content. Figure 1c shows the tensile strength and elongation of aluminum matrix composites with nanometer TiB_2_ only. With an increase in nanometer TiB_2_, the tensile strength first increases and then decreases, and the elongation reaches its maximum when 4% nanometer TiB_2_ is added. The optimal tensile strength can reach up to 187 MPa.

### 2.2. Sample Fabrication Method for Double-Scale TiB_2_/(6061Al + Ti) Composites

**Figure 2:**
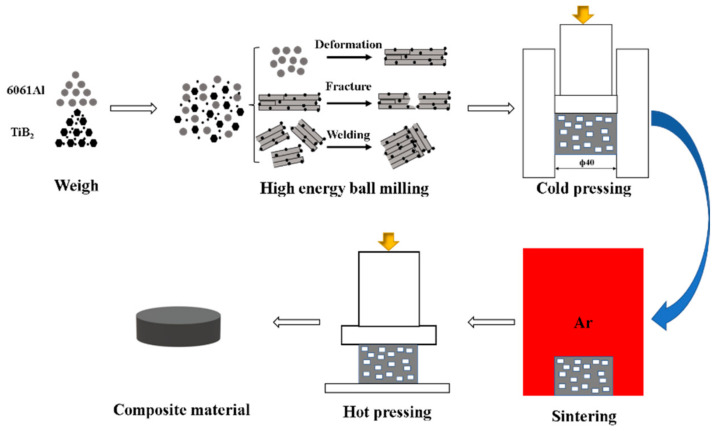
Experimental flow chart.

To determine the optimum amount of TiB_2_ nanoparticles to be added, aluminum matrix composites were prepared with TiB_2_ nanoparticle content of 0, 2 wt.%, 4 wt.%, and 6 wt.%, as shown in Table 3. First, the micron TiB_2_ was weighed with mass fractions of 0, 1 wt.%, 3 wt.%, and 5 wt.%, and the nano TiB_2_ content was set at 4 wt.%. As shown in Table 4, the micron and nano TiB_2_ were mixed and ultrasonicated with anhydrous ethanol for 2.5 h to improve the agglomeration of reinforcing body powder. To prevent overheating during the powder reaction, intermittent ultrasonication was performed (every 0.5 h, with 150 mL anhydrous ethanol added every 15 min). After that, the mixed TiB_2_ and 6061Al were loaded into a ball milling jar, and then Ti powder with a mass fraction of 3 wt.% was weighed and added. Wet grinding was carried out using a QM-3SP4 planetary ball mill (Nanjing Nanda Instrument Co., Ltd., Nanjing, China) in an argon environment, with a rotational speed of 280 r/min, time of 12 h, and ball material ratio of 10:1 to prevent excessive cold welding. The process was paused for 15 min for every 72 min of ball milling. After ball milling, vacuum drying was performed for 4 h. The powder was then subjected to a second dry milling with added stearic acid as a process control agent, at the same speed, time, and ball feed ratio as the first wet milling. The powder was obtained and subsequently weighed in a vacuum glove box. Figure 2 shows a flow chart describing the experimental preparation.

**Figure 3:**
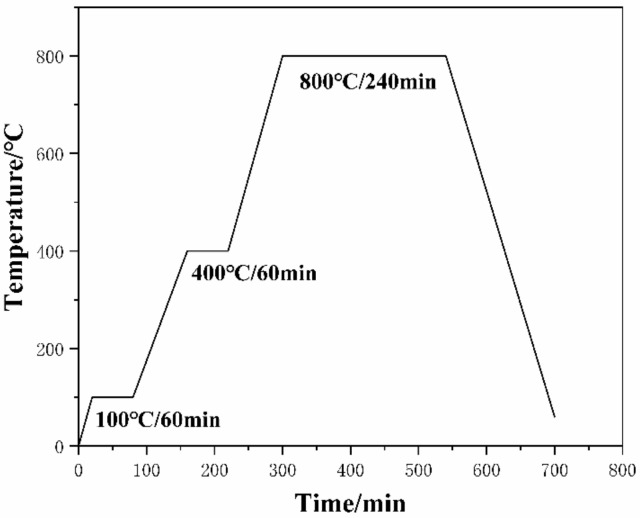
Sintering process curve.

Then, using a four-column hydraulic press (YLX32-100, Jiangsu Longxu Heavy Industry Machinery Co., Ltd., Nantong, China), the ball-milled powder was sequentially cold pressed (pressure of 425 MPa, holding time of 6 min) to obtain a cylindrical billet and then sintered in an argon atmosphere using a tube sintering furnace (TF1-1200, Verder Shanghai Instruments and Equipment Co., Ltd., Shanghai, China) with a temperature increase rate of 5 °C/min. The sintering temperature curve is shown in Figure 3. Firstly, the temperature was raised to 100 °C and maintained for 60 min to remove any water vapor. After that, it was raised again to 400 °C and maintained for 60 min to remove the process control agent stearic acid, and finally raised to 800 °C, which was selected for the sintering. After sintering, the block material was cooled to room temperature in the furnace, and then hot-pressed using a hydraulic press (temperature of 520 °C, pressure of 525 MPa, holding time of 10 s). Finally, T-6 heat treatment was performed.

### 2.3. Microstructure and Property Characterization of Double-Scale TiB_2_/(6061Al + Ti) Composites

Microstructural characterization and the composition of the specimens’ wear were determined using a scanning electron microscope (SEM, SU8010, Hitachi Limited, Tokyo, Japan). An X-ray diffractometer (XRD, Empyrean, PANalytical B.V., Almelo, The Netherlands, scanning speed 10°/min, scanning angle 10–90°, Cu-K) was used for analysis.

The tensile test was conducted using a universal tensile testing machine (CTM9200, MTS SYSTEMS(CHINA) Co., Ltd., Shanghai, China) with a tensile rate of 0.2 mm/min. The total length of the tensile specimen was 30 mm and the marking distance was 10 mm. The nanoindentation properties were characterized via a nanoindentation test (UML VMHT, Walter Uhl technische Mikroskopie GmbH & Co. KG., German), with a loading force of 50 g and a holding time of 10 s, and the average of the 10 test values for each specimen was taken as the final test result. The friction and wear tests were carried out using a high-speed reciprocating friction and wear tester (MFT-EC4000, Lanzhou Huahui Instrument Technology Co., Ltd., Lanzhou, China). The friction partner was a GGr15 steel ball with a diameter of 6 mm, and the sliding time, distance, and frequency of the applied loads were 20 min, 5 mm, 2 Hz, and 20 N, respectively, according to Formula (1), which was used to calculate the volumetric wear rate of the composite material ω:

ω = V/SP(1)

where V represents the wear volume in mm^3^; S represents the sliding distance in m; P represents the applied normal load in N.

The 3D shape of the abraded surface, the cross-sectional profile, and the wear volume were tested using an optical profilometer (Bruker Contour GT-K 3D, Bruker, Ettlingen, Germany).

## 3. Results and Analysis

### 3.1. Microstructure and Phase Analysis of Powder after Ball Milling

**Figure 4:**
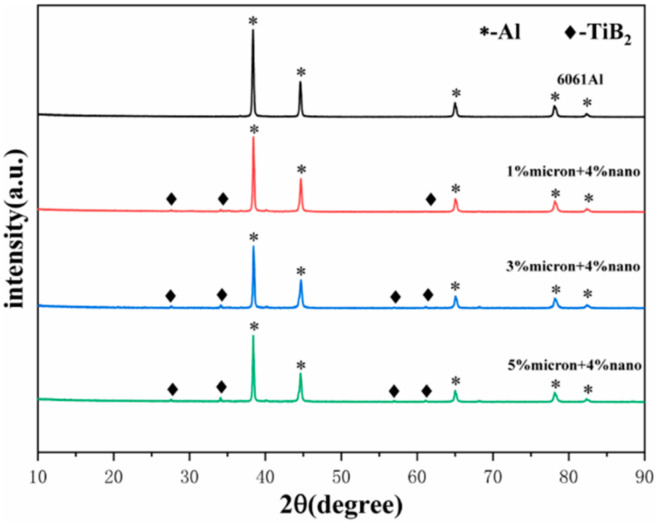
XRD spectra of powder after ball milling.

**Figure 5:**
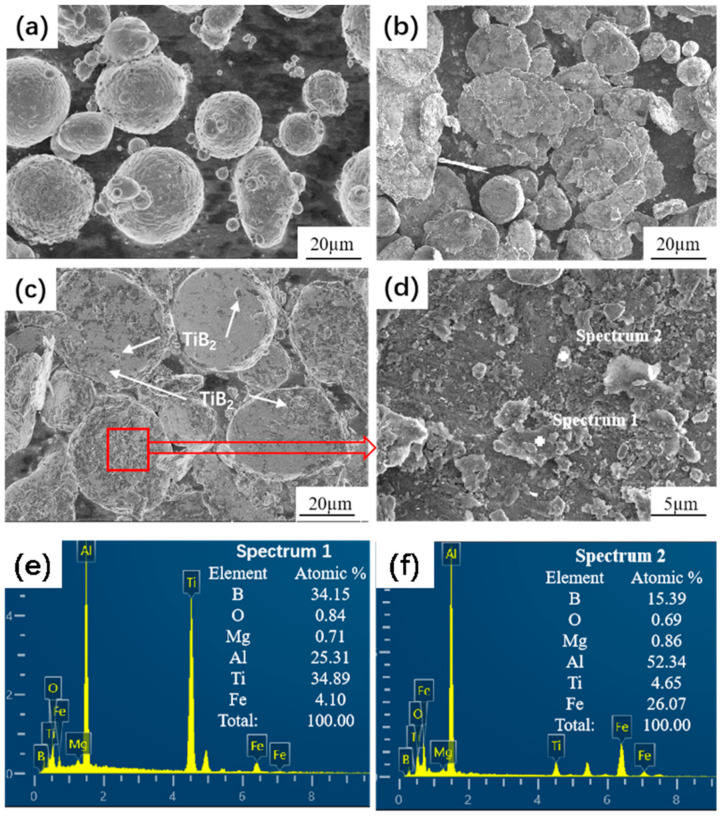
SEM images of powders after ball milling (**a**,**c**) with and (**b**,**d**) without enhancer. (**e**,**f**) The corresponding EDS point scanning.

Figure 4 shows the XRD patterns of the powder after ball milling. Al and TiB_2_ peaks occurred as a result of additions of 5% (1% micron + 4% nano), 7% (3% micron + 4% nano), and 9% (5% micron + 4% nano) of TiB_2_. No other substances were produced, indicating that the powder did not react during the high-energy ball milling process. During the high-energy ball milling process, the matrix particle shape changed from spherical to flaky (Figure 5a,b), which is conducive to the dispersion of the enhancer. Meanwhile, due to the cold welding phenomenon, the powder particles repeatedly experienced fracture and breakage, cold welding, and reorganization, and some of the reinforcement particles were embedded in or surrounded by the flaky 6061Al particles; see Figure 5c,d. The EDS spot scanning is shown in Figure 5e,f. The trace elements contain Al and 6061Al. Elements of B and Ti are detected as well, and the element of B corresponds to the enhancer TiB_2_. In the Figure, the Ti element is obviously more intense than the B element peak, suggesting that the Ti element content is higher than that of B element. The results also confirm that the material wrapped and inlaid on the aluminum matrix particles is Ti powder and TiB_2_ enhancer. The XRD spectra of the powder after ball milling show that no other substances, such as oxides, were detected, indicating that no other harmful reactions occurred during the ball milling process.

### 3.2. The Role of Ti in Double-Scale TiB_2_/(6061Al + Ti) Composites

#### 3.2.1. Composite Powder Morphology after Addition of Ti

**Figure 6:**
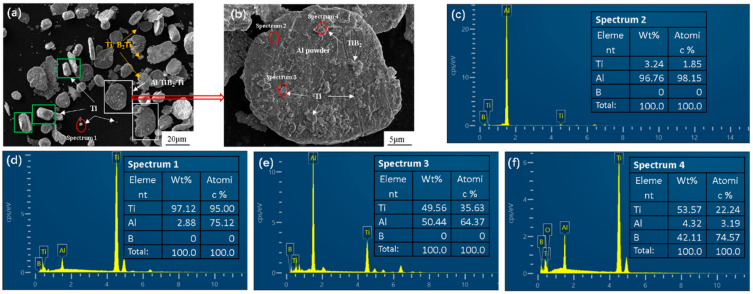
SEM images of composite powders containing 3% Ti. (**a**) Overall overview image; (**b**) enlarged image of single particle; (**c**–**f**) the corresponding EDS point scanning.

Figure 6a shows the composite powder containing Ti. After ball milling, the powder was divided into three major categories: irregular flakes (Figure 6a white rectangle); irregular, short thick rod (Figure 6a green rectangle); and some small debris. The EDS point scanning confirmed that the short rod particles and small debris were Ti particles. This is because high-energy ball milling produces a huge amount of energy. The Al is soft, and it will undergo strong plastic deformation as it flakes. TiB_2_ is a high-melting-point, high-hardness ceramic particle. It is unaffected by high-energy ball milling, though some large Ti particles around the corners may be broken into small chips, and its overall configuration does not change as much as Al. Figure 6b shows that the particles embedded in the Al matrix do not exhibit clear differences in appearance, while Figure 6c–f shows the EDS point scan, which confirms that the particles embedded in the Al matrix contain TiB_2_ as well as Ti. The size of the original Ti powder added in the present study is 40–50 μm, which is larger than the original Al powder particles. This indicates that the Ti embedded in the Al matrix is crushed by the large Ti particles obtained during the high-cooling ball milling. The cold welding causes Ti to adhere to the surface of large flaky Al particles or attach to TiB_2_, thus improving the dispersibility of the Ti, which is attached to TiB_2_. As such, it will act as a medium for connecting the interface of the Al matrix with the reinforcing body.

#### 3.2.2. Microstructure of Ti in Bulk Composites

**Figure 7:**
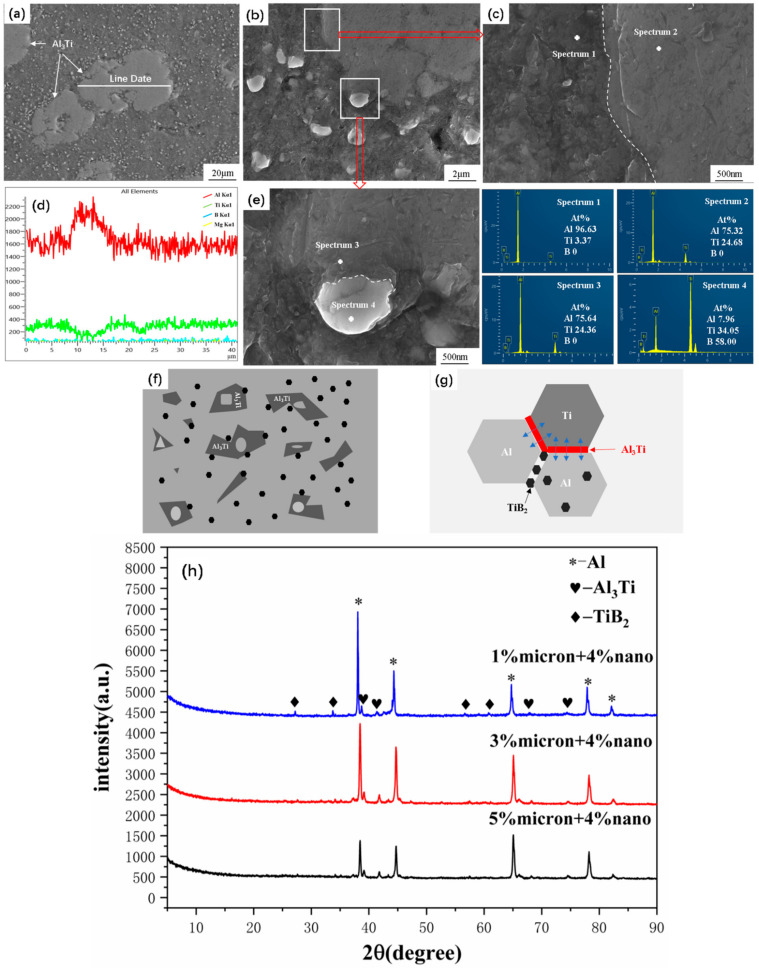
SEM images/EDS energy spectra of composites containing 3% Ti. (**a**,**d**) Line scanning; (**b**–**e**) interface magnification; (**f**,**g**) schematic of Al-Ti- TiB_2_ interface; (**h**) XRD spectra of TiB_2_/(6061Al + Ti) composites.

Figure 7 shows the microstructure of the added Ti bulk composite. In Figure 7a, a second phase with a size of 20–60 µm was observed. The corresponding EDS line scan results in Figure 7a,d suggest that most of the particles have an X-ray intensity ratio of Al and Ti of roughly 3:1. The Ti-Al phase diagrams illustrate a reaction between Al and Ti, which prompts the generation of intermetallic compounds such as Al_3_Ti, Al_2_Ti, AlTi_3_, and AlTi. The EDS spot scan results in Figure 7c,e, combined with the XRD spectra of TiB_2_/(6061Al + Ti) composites in Figure 7h, confirm in situ synthesis of Al_3_Ti. Two factors contributed to the reaction. One of them is that after the high-energy ball milling mentioned earlier, the sustained and strong shear force of the milling ball induced plastic deformation. This caused Ti to break into fine particles and adhere to Al and promoted the atomic diffusion and Ti-Al interface reaction. Secondly, the sintering temperature of this experiment reached 800 °C and the sintering time lasted up to 240 min, which further promotes the reaction between Ti and Al. It can also be seen from Figure 7b that the large particles of Al_3_Ti were not intact. When the particle sizes exceeded a certain level, a gap was observed after sintering and hot-pressing. Figure 7d further demonstrates that the intermediate gap portion of Al_3_Ti contains Al. Figure 7c,e show EDS point scans, including magnified images of the interfaces of Al_3_Ti/Al and Al_3_Ti/TiB_2_. Both interfaces are clear and smooth with good interfacial bonding. The huge amount of energy generated during the sintering process between Al_3_Ti and TiB_2_, as well as the ball milling, which resulted in certain defects on both sides, caused the atoms between the two to diffuse with each other and firmly bond together. These TiB_2_ particles, which firmly bonded to the in situ-formed Al_3_Ti, were locked in place by its stable Ti-Al covalent bond. Figure 7f,g shows the distribution and interfacial bonding schematic of Al, Ti, and TiB_2_ tissues. The addition of Ti caused the in situ synthesis of Al_3_Ti, improving the dispersion of the reinforcement TiB_2_ and effectively helping the load transfer to the reinforcement.

### 3.3. Microstructure and Phase Analysis of Bulk Materials after Sintering and Hot-Pressing

**Figure 8:**
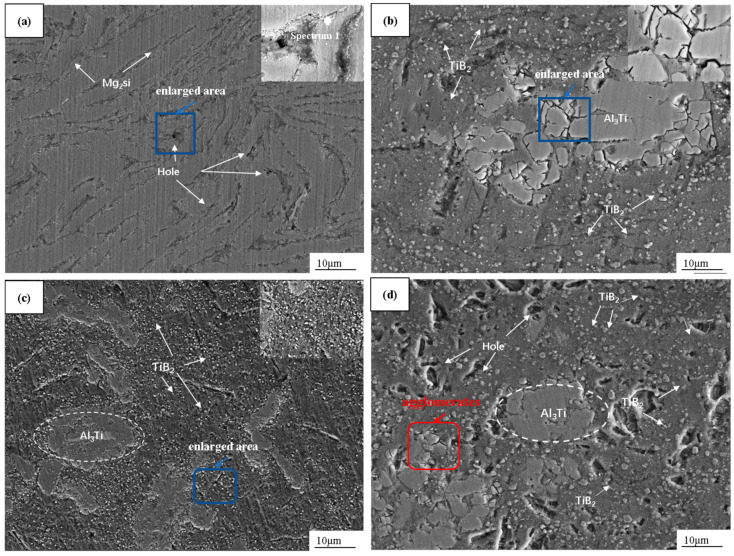

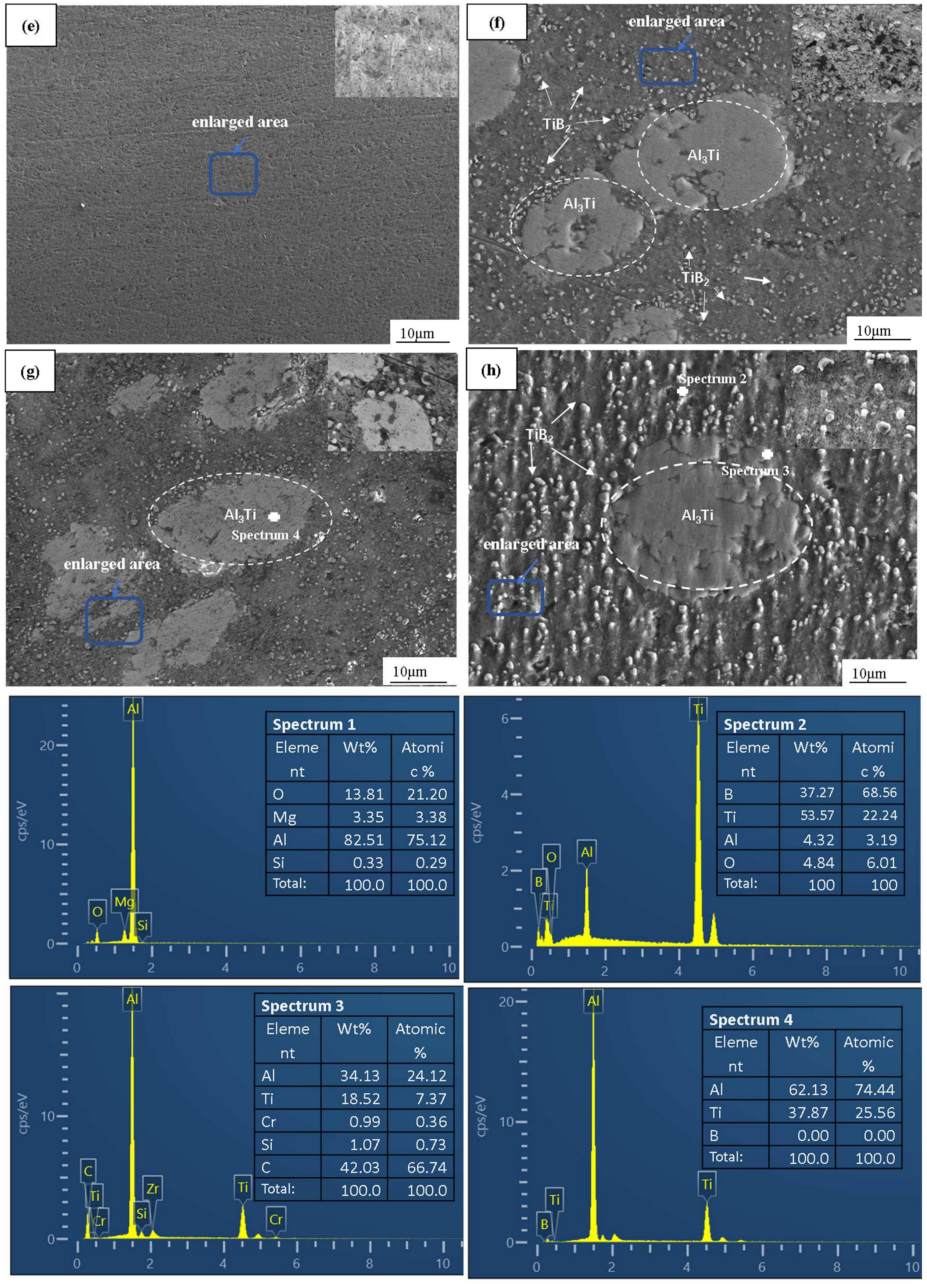
SEM images of dual-scale TiB_2_/6061Al composites. (**a**–**d**) Sintered state 0; 1% micron + 4% nano; 3% micron + 4% nano; 5% micron + 4% nano. (**e**–**h**) Hot-pressed state 0; 1% micron + 4% nano; 3% micron + 4% nano; 5% micron + 4% nano.

Figure 8 shows SEM images of the bulk composites. Figure 8a–d show the sintered state, in which numerous pore defects can be observed, while Figure 8e–h show the hot-pressing state. An improvement in pore defects was observed after the hot-pressing. The red rectangular box in Figure 8d highlights the excessively large agglomerates of TiB_2_ reinforcement content. These aggregated particles do not have a metallurgical bonding interface, affecting the stress concentration and tensile test when the load is applied. These aggregated particles are not involved in the load transfer effect, which is the source of the failure of the material and has a serious impact on the mechanical properties of the material. The large irregular particles shown in the dashed box of the ellipse were confirmed to be Al_3_Ti via EDS spot scanning, and the small particles with uniform distribution in the figure were confirmed to be TiB_2_ particles. An enlarged view of the blue rectangular box is shown in the upper right corner, and the poor interfacial bonding between Al_3_Ti/micron TiB_2_ as well as the Al_3_Ti/Al matrix can be clearly seen in Figure 8b,c. However, such interfaces do not exist in Figure 8f,g; the interfacial bonding is significantly smoother and the pores and cracks are significantly reduced after hot-pressing. The addition of TiB_2_ particles prevents the matrix from growing during sintering, which will help to refine the grains. The TiB_2_ particles are more uniformly distributed due to the high temperature of the hot-pressing. The pressure ambiently flows to the better plasticized 6061Al matrix, enabling a more uniformly dispersed distribution of the particles [34]. The enlarged area in Figure 8f–h shows that the TiB_2_ particles are not in a plane with the substrate, and all the particles are in a slightly convex state. This is because the 6061Al substrate is softer, while the TiB_2_ particles are hard for the ceramic particles. The substrate is abraded for an extended period of sanding and polishing, but there is no observation that any particles are removed during this process, which reflects the strong bond between the particles and the substrate. A large amount of Mg_2_Si is observed to precipitate at the grain boundary in Figure 8a, while the precipitated phase melts back into the matrix in Figure 8e. The reinforced composites do not produce Mg_2_Si either in the sintered or hot-pressed state, as TiB_2_ inhibits the precipitation of Mg_2_Si.

### 3.4. Mechanical Property of Double-Scale TiB_2_/(6061Al + Ti) Composites

**Figure 9:**
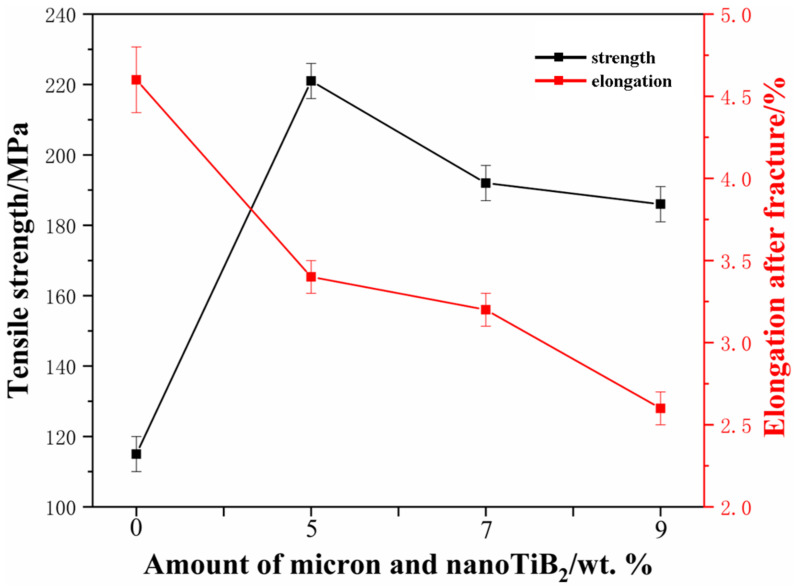
Tensile strength and elongation at break of dual-scale TiB_2_/6061Al composites.

Figure 9 shows the tensile strength and elongation of biscaled TiB_2_/6061Al composites with different TiB_2_ content. The tensile strength initially increases and then decreases with the increase in TiB_2_ content, and the elongation after the break shows a continuous decreasing trend. Compared with pure 6061Al, the tensile strength of all the TiB_2_-added reinforcements are significantly improved; the TiB_2_ content of 5% (1% micron + 4% nano) exhibits the largest (221 MPa) increase in tensile strength, and it is 93.9% higher than that of pure 6061Al, while the elongation at break is 3.3%, which is slightly lower than that of pure 6061Al. TiB_2_ composites with 5% (1% micron + 4% nano) show the best tensile properties, and the increase in strength is attributed to the good interfacial bonding of the biscaled TiB_2_ particles with the Al matrix, the efficient transfer of the load from the matrix to the particles during the tensile test, the mismatch of the coefficients of thermal expansion of TiB_2_ and the matrix, the large number of dislocations in the matrix during the cooling process of the heat deformation, and the reinforcing effect of the in situ-synthesized Al_3_Ti.

**Figure 10:**
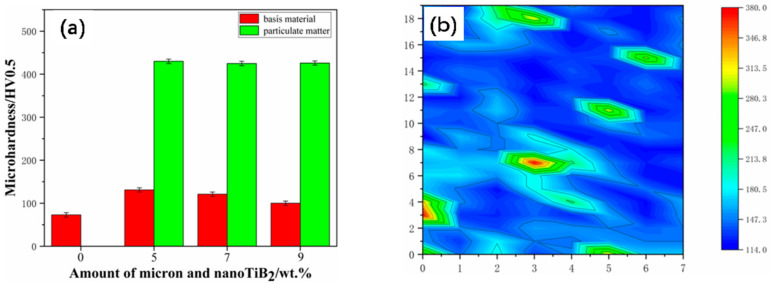
Hardness of double-scale TiB_2_/6061Al composites. (**a**) Hardness hitting plot; (**b**) hardness distribution plot.

Figure 10 shows the results of the double-scale TiB_2_/6061Al microhardness test, while Figure 10a shows the microhardness of double-scale TiB_2_/6061Al composites with different contents. The hardness (pm) on the TiB_2_ particles is 430 HV, and the hardness (bm) on the matrix is obviously improved by the addition of the TiB_2_ particle reinforcement. The hardness (bm) on the matrix for TiB_2_ content of 5% (1% micron + 4% nano), 7% (3% micron + 4% nano), and 9% (5% micron + 4% nano) was 131 HV, 121 HV, and 101 HV, respectively, which was 79.5%, 65.8%, and 38.4% higher than the hardness of the pure 6061 matrix (73 HV). This can be attributed to the difference in deformation resistance between the Al matrix and micron TiB_2_ particles. A large amount of stress residue can be generated at the interface between the Al matrix and micron TiB_2_ particles after hot-pressing, resulting in the formation of a particle deformation zone (PDZ). Figure 10b shows the hardness distribution of composites with TiB_2_ content of 5% (1% micron + 4% nano). The closer the particles of the matrix, the higher the hardness. Moreover, the distribution of the reinforcement is also uniform; this may explain the mismatch between the coefficients of thermal expansion of TiB_2_ and the matrix, which results in a large number of dislocations being generated in the matrix. TiB_2_ particles also prevent dislocation motion to enhance the material’s resistance to deformation, which is ultimately reflected by an increase in hardness. The addition of TiB_2_ particles refines the matrix grains, increases the number of grain boundaries, and hinders the dislocation slip, which further improves the hardness [35].

### 3.5. Room Temperature Friction and Wear Performance Analysis of Double-Scale TiB_2_/6061Al Composites

**Figure 11:**
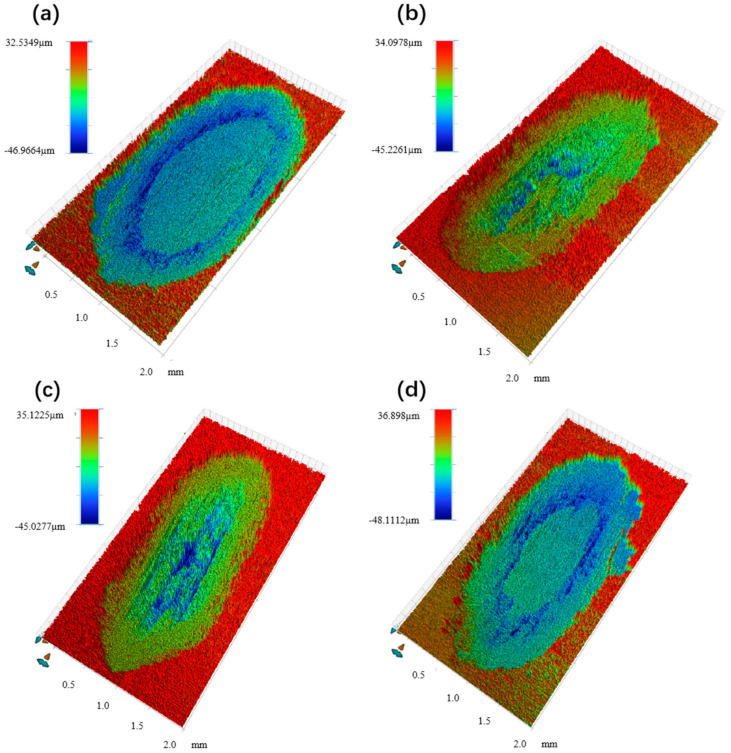
Surface topography of 3D abrasion marks of composites with different TiB_2_ contents. (**a**) 0; (**b**) 1% micron + 4% nano; (**c**) 3% micron + 4% nano; (**d**) 5% micron + 4% nano.

**Figure 12:**
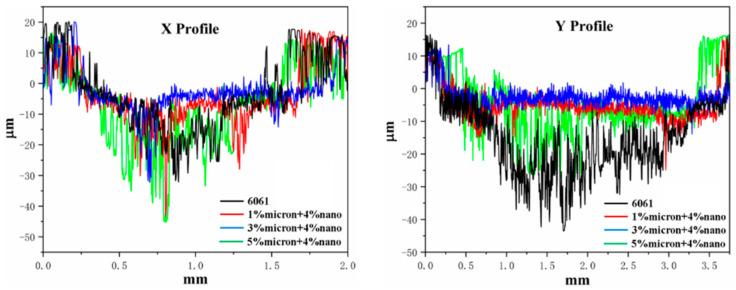
Cross-section profile curves corresponding to wear marks of composites with different TiB_2_ content.

Figure 11 shows the three-dimensional abrasion mark morphology of composites with different TiB_2_ contents. Figure 12 shows the contour curves of the cross-section at the level x and normal y of the abrasion mark. From Figure 11, it can be seen that when the TiB_2_ content is 5% (1% micron + 4% nano) and 7% (3% micron + 4% nano), the middle section of the composite abrasion marks is roughly rectangular, though it appears semicircular at both ends, and the composite abrasion marks with a TiB_2_ content of 9% (5% micron + 4% nano) are roughly the same as those of the pure 6061Al matrix, which have an elliptical shape. Combined with Figure 12, it can be noted that the width and depth of the pure 6061Al matrix are the largest. With the increase in TiB_2_, the width and depth gradually decrease. When the TiB_2_ content is 7% (3% micron + 4% nano), the cross-sectional profile curves in the x and y directions are more homogeneous, and the abrasion mark depth and width are the smallest. Anomalous changes occur when the TiB_2_ content is increased to 9% (5% micron + 4% nano), with the cross-sectional profile curves in the x and y directions becoming more buoyant, and the width and depth of the abrasion marks increasing.

**Figure 13:**
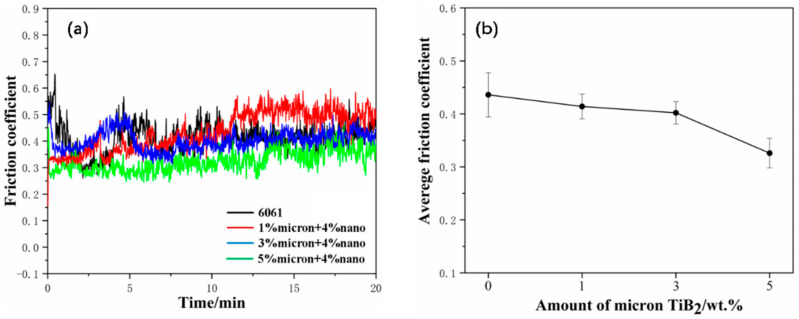
Friction coefficient versus time curve and average friction coefficient of composites with different TiB_2_ content. (**a**) Friction coefficient versus time curve. (**b**) Average friction coefficient.

The coefficient of friction and wear rate are important indicators of the friction and wear performance of composites. As shown in Figure 13a, the friction coefficient stabilizes after the addition of double-scale TiB_2_ compared to the pure 6061Al matrix, with an overall gradual increasing trend. This is due to the fact that TiB_2_ is a high-hardness ceramic particle. Adding it to the matrix improves the material’s hardness, as well as its ability to resist plastic deformation. This leads to a reduction in the average coefficient of friction of the material and stabilization of the change in this coefficient. At the beginning of the friction experiment, the hard particles will play a supporting role and reduce the contact area between the wear parts and the friction pair. Then, as the experiment continues, the depth of the abrasion marks will increase, and there will be a gradual increase in the contact area, resulting in an overall increase in the friction curve [36]. Combined with Figure 13b, the friction coefficient decreases with the increase in TiB_2_, and the average friction coefficient displays the highest decrement when the content is 9% (5% micron + 4% nano). Within a certain range, the more reinforcing phase content added, the greater the presence of hard particles that can play a supporting role, meaning that the material friction coefficient reduction will be more obvious. The coefficient of friction decreases dramatically at a content of 9% (5% micron + 4% nano), indicating that when the content reaches a certain value, it is poorly bonded to the substrate, and can act as a lubricant in friction and wear experiments. Table 5 shows the wear volume and volumetric wear rate of composites with different TiB_2_ content. Composites with TiB_2_ content of 7% (3% micron + 4% nano) have higher average coefficient of friction and the lowest volumetric wear rate of 0.402 and 0.216 mm^3^∙N^−1^∙m^−1^.

### 3.6. Room Temperature Frictional Wear Mechanisms of Double-Scale TiB_2_/6061Al Composites

**Figure 14:**
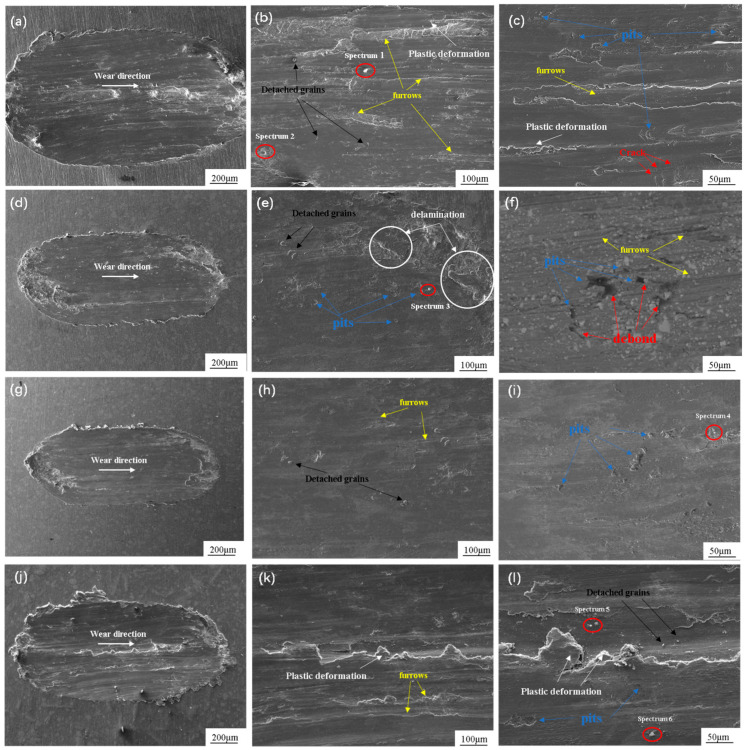

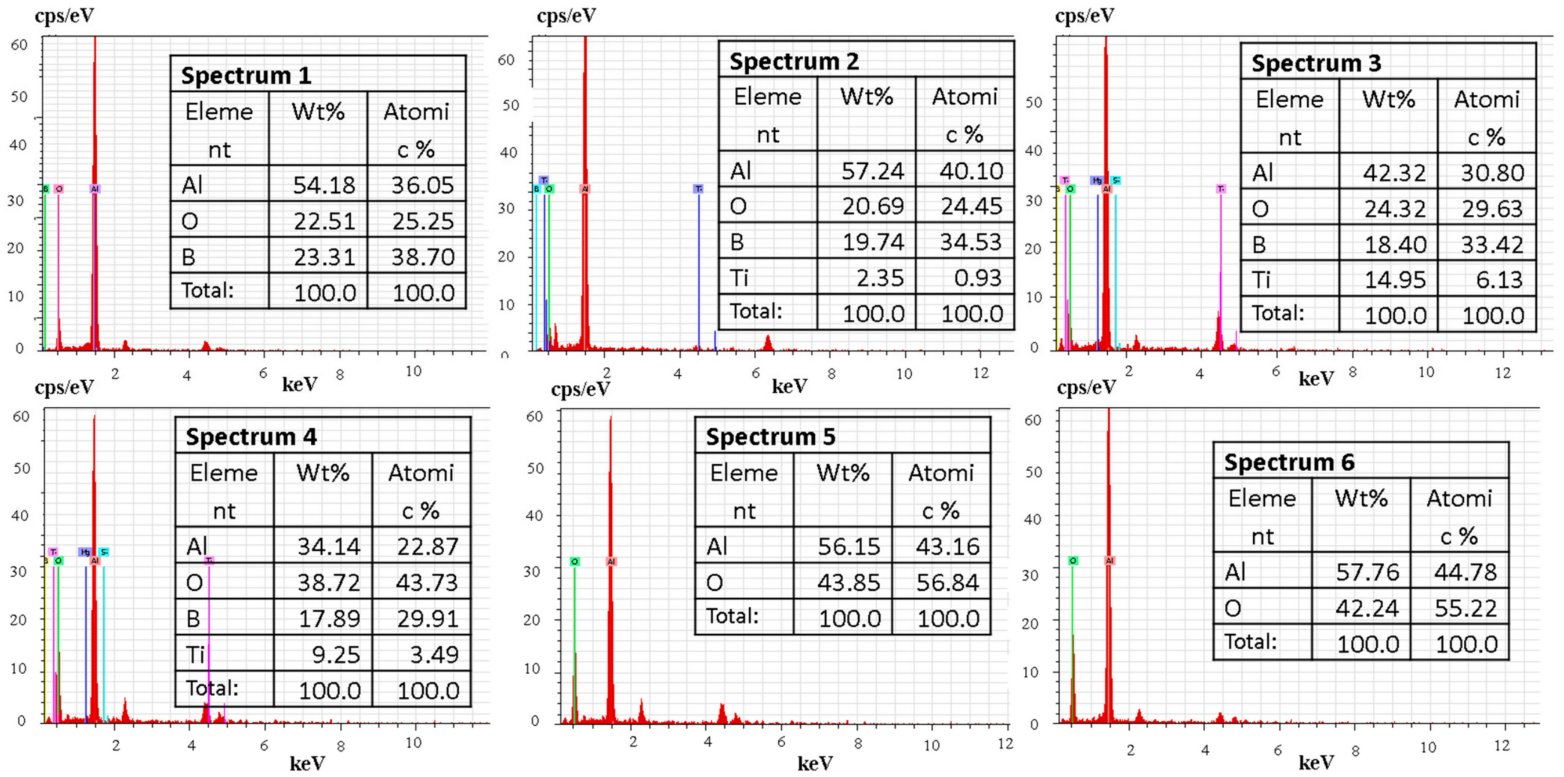
Abrasion morphology of composites with different TiB_2_ contents. (**a**–**c**) 0; (**d**–**f**) 1% micron + 4% nano; (**g**–**i**) 3% micron + 4% nano; (**j**–**l**) 5% micron + 4% nano.

Figure 14 shows the abrasion scar morphology of composites with different TiB_2_ content. Figure 14a shows that the abrasion scar is very wide, with obvious bright white areas. These bright white areas represent oxides produced during the friction wear experiments. Figure 14b,c exhibit a large number of furrows and significant delamination. This is because the initial stage of friction in the matrix at the point of contact produces a solid-phase welding, that is, adhesion. As the relative motion continues, the adhesion point is sheared, and then continues to adhere, and then shear, in the surface of the material, creating varying degrees of plough grooves and craters [36,37], in line with the characteristics of the adhesive wear. Due to the continued exposure to friction, the substrate surface of the oxidized layer will be destroyed. The newly exposed Al substrate continues to experience friction due to the energy generated by the oxygen in the air, which produces a new oxidized layer. Additionally, the constant process of destruction and generation causes bright white oxide particles or oxide folds until the end of the test, confirming that the wear mechanism is accompanied by oxidative wear. Figure 14d–i show that the furrows change from wide and deep to thin and shallow compared to the pure matrix in the composite. The surface is relatively flat, as the TiB_2_ reinforcement inhibits the plastic deformation of the matrix. The continued friction leads to a loss of TiB_2_ particles, resulting in debonding and pit formation. Debonded particles adhere to the friction pair, causing the substrate to produce greater shear. With this increase in shear, a critical value is reached; some of the particles are detached from the friction pair and are embedded in the material to form a pile-up and peeling. The arrow and rectangular box point out the pits and pile-up, as a result of the friction mechanism from the adhesive wear to abrasive wear. In Figure 14f, a small amount of oxides is observed at the end of the abrasion mark, which indicates that the wear mechanism is still accompanied by slight oxidative wear when the TiB_2_ content is 5% (1% micron + 4% nano), and abrasive wear occurs when the TiB_2_ content is 7% (3% micron + 4% nano). Figure 14j–l show the morphology of the abrasion marks with TiB_2_ content of 9% (5% micron + 4% nano), at which time the abrasion marks as a whole become wide and deep, as shown in Figure 14j, and numerous very deep and wide furrows and craters are observed. The reason for this is that an excess of TiB_2_ content leads to a loose substrate structure, and a larger number of micrometer-sized particles are detached to form abrasive particles between the friction pair and the substrate. These abrasive particles, caused by the shear force, also produce greater damage to the substrate, increasing the amount of abrasive wear.

## 4. Conclusions

Double-scale particle-reinforced TiB_2_/6061 aluminum matrix composites were prepared via two high-energy ball milling methods. Powder metallurgy was used to alter the structure of the powder, which became flaky as a result. The reinforcement particle was embedded in or wrapped by the matrix and uniformly dispersed within the material. Strong and dense interface bonding composites were obtained after sintering and hot-pressing.

The micro–nano synergistic reinforcement and the in situ-synthesized Al_3_Ti reinforcing phase play significant roles in the TiB_2_ composite material with 1% micron + 4% nano + 3% Ti. The tensile strength and hardness of the composites reached 221 MPa and 131 HV_0.05_, respectively, showing increases of 93.9% and 79.5% compared with the pure matrix material created according to the same process. The addition of TiB_2_ reinforcement effectively reduces the coefficient of friction and volumetric wear rate and significantly improves the wear resistance of the composites. The composites with a TiB_2_ content of 7% (3% micron + 4% nano) show optimal friction and wear properties, with high average coefficients of friction and the lowest volumetric wear rates of 0.402 and 0.216 mm^3^∙N^−1^∙m^−1^, respectively. The friction mechanism changes from adhesive wear with slight oxidative wear to abrasive wear.

## Figures and Tables

**Table 1:** Chemical composition of 6061 aluminum alloy (wt.%).

Mg	Mn	Zn	Cr	Ti	Si	Fe	Al
1.15	0.14	0.2	0.1	0.14	0.6	0.7	Bal.

**Table 2:** Chemical composition of TiB_2_ (wt.%).

Ti	B	N	Si	Fe	Ni
>65.45	>29.55	<0.09	<0.14	<0.08	<0.01

**Table 3:** Experimental schedule of nano-scale TiB_2_/6061Al composites.

Experiment Number	Micron TiB_2_/wt.%	Nano TiB_2_/wt.%
1	0	0
2	0	2
3	0	4
4	0	6

**Table 4:** Experimental schedule of double-scale TiB_2_/(6061Al + Ti) composites.

Experiment Number	Micron TiB_2_/wt.%	Nano TiB_2_/wt.%	Ti/wt.%
1	0	0	0
2	1	4	3
3	3	4	3
4	5	4	3

**Table 5:** Wear volume and volume wear rate of the composites with different TiB_2_ contents.

ω(TiB_2_)/%	0	1	3	5
Wear volume/mm^3^	4.51 × 10^−2^	2.52 × 10^−2^	2.16 × 10^−2^	2.96 × 10^−2^
Volume wear rate/(mm^3^∙N^−1^∙m^−1^)	0.451	0.252	0.216	0.296

## Data Availability

Data are contained within the article.
